# Examining Intersectionality and Barriers to the Uptake of Video Consultations Among Older Adults From Disadvantaged Backgrounds With Limited English Proficiency: Qualitative Narrative Interview Study

**DOI:** 10.2196/65690

**Published:** 2025-01-06

**Authors:** Laiba Husain, Trisha Greenhalgh

**Affiliations:** 1 Department of Primary Care Health Sciences University of Oxford Oxford United Kingdom

**Keywords:** digital health disparities, video consultations, intersectionality, health inequity, digital capital, mobile phone

## Abstract

**Background:**

The rapid shift to video consultation services during the COVID-19 pandemic has raised concerns about exacerbating existing health inequities, particularly for disadvantaged populations. Intersectionality theory provides a valuable framework for understanding how multiple dimensions of disadvantage interact to shape health experiences and outcomes.

**Objective:**

This study aims to explore how multiple dimensions of disadvantage—specifically older age, limited English proficiency, and low socioeconomic status—intersect to shape experiences with digital health services, focusing on video consultations.

**Methods:**

Following familiarization visits and interviews with service providers, 17 older people with multiple markers of disadvantage (older age, low socioeconomic status, and limited English proficiency) were recruited in the Redbridge borough of London. Data collection included narrative interviews and ethnographic observations during home visits. Field notes captured participants’ living conditions, family dynamics, and technological arrangements. Guided by intersectionality theory and digital capital concepts, interviews explored participants’ experiences accessing health care remotely. Intersectional narrative analysis was used to identify key themes and examine how different forms of disadvantage interact. We developed theoretically informed narrative portraits and user personas to synthesize findings.

**Results:**

Analysis revealed that the digitalization of health care can exacerbate existing inequities, erode trust, compound oppression, and reduce patient agency for multiply disadvantaged patient populations. Examining intersectionality illuminated how age, language proficiency, and socioeconomic status interact to create unique barriers and experiences. Key themes included the following: weakened presence in digital interactions, erosion of therapeutic relationships, shift from relational to distributed continuity, increased complexity leading to disorientation, engagement shaped by previous experiences of discrimination, and reduced patient agency.

**Conclusions:**

This study provides critical insights into how the digitalization of health care can deepen disparities for older patients with low income and limited English proficiency. By applying intersectionality theory to digital health disparities, our findings underscore the need for multifaceted approaches to digital health equity that address the complex interplay of disadvantage. Recommendations include co-designing inclusive digital services, strengthening relational continuity, and developing targeted support to preserve agency and trust for marginalized groups in an increasingly digital health care landscape.

## Introduction

### Overview

While existing research has explored digital health disparities [1-5] and the application of intersectionality theory in health care [6-8] separately, there remains a critical gap in understanding how multiple dimensions of disadvantage intersect specifically in the context of digital health services. Previous studies have largely focused on single-axis analyses of digital exclusion [9-11]. For example, Ramsetty and Adams [9] examined socioeconomic status as a determinant of digital health access through quantitative analysis of technology ownership and internet connectivity rates. Eberly et al [10] used a large-scale quantitative study to analyze telemedicine access solely through the lens of age, finding lower video visit completion rates among older adults. Similarly, Donaghy et al [11] investigated the acceptance of video consultations primarily through the single dimension of technological literacy using survey data. While these quantitative studies provide valuable insights into individual factors, they have not adequately examined the compounded effects of age, language proficiency, and socioeconomic status on experiences with digital health technologies, particularly video consultations. Furthermore, there is a lack of in-depth, qualitative research that captures the lived experiences of multiply disadvantaged patients navigating these digital health spaces [12]. This study aimed to address this gap by applying an intersectional lens to explore how various forms of disadvantage interact and manifest in the context of video consultations, providing a more nuanced and comprehensive understanding of digital health disparities among susceptible populations.

We adopted an intersectional perspective from feminist studies to highlight the intersection and entanglement between digital technology, structural stratifications, and ingrained tendencies of “othering” in societies. This approach allowed us to move beyond simplistic notions of digital divisions to examine how digital technology is implicated in complex and intersectional systems of power. Drawing on narrative interviews with older, low-income individuals with limited English proficiency, we examined intersectionality and how video consultations can exacerbate existing inequities for multiply disadvantaged patient populations.

Our analysis revealed that digital health disparities operate at the intersection of multiple fracture lines of difference that mediate various spaces of inclusion and exclusion. We argue that addressing digital health disparities requires moving beyond single-axis analyses to consider how different aspects of disadvantage intersect in individual lives. This paper contributes to information systems (ISs) literature by providing a richer theorization of digital inequity, highlighting the need for intersectional approaches to digital health equity. We propose a research agenda that calls for IS scholars to reconceptualize actors beyond simplistic notions of “users,” to consider positioning rather than contextualizing, and to examine how digital health technologies are intertwined with producing and reproducing social orders and stratifications. Our findings have important implications for policy and practice in designing and implementing more inclusive and equitable digital health services.

### Background and Literature Review

#### Overview

In the United Kingdom, video consultation uptake has increased significantly since the COVID-19 pandemic. National Health Service (NHS) England reported that before the pandemic, <1% of primary care appointments used video, but this number rose sharply during early 2020 to approximately 13% as part of the NHS Long Term Plan to expand digital-first care [13]. By 2023, about 9% of consultations in general practice continued to use video, reflecting an enduring shift toward remote health care delivery despite a return to face-to-face appointments as the dominant mode (65%) [14,15]. However, the use of video consultations varies significantly across demographic groups. Research indicates that older patients (aged >65 years) are less likely to use video services, with adoption rates around 7%, compared to 20% among younger adults aged between 18 and 34 years. Similarly, individuals from lower socioeconomic backgrounds and those with limited English proficiency face notable barriers, demonstrating uptake rates <10% [14,16].

#### Digital Health Inequalities and the Digital Divide

The IS literature has long engaged with the concept of the digital divide, traditionally focusing on accessibility, literacy, and adoption of digital technologies [17]. This discourse often conceptualizes individuals as “users” of technology, assigning them to specific group categories such as “the excluded” or based on binary divisions of “haves” and “have-nots” [18]. However, this notion of the digital divide fails to account for the multifaceted and compounded nature of digital inequality [12,19]. Recent scholarship has recognized that digital exclusion is a complex and dynamic phenomenon influenced by various factors beyond access to technology, including age, gender, education, and socioeconomic status [20-25]. As health care becomes increasingly digitalized, there is a growing need to understand how these broader digital inequalities manifest in health contexts. The concept of digital health equity has emerged as a critical area of study, examining how social determinants of health intersect with digital access and skills to shape health outcomes. This evolving field calls for more nuanced, intersectional approaches to understanding and addressing disparities in digital health access and use.

#### Intersectionality Theory

To address the limitations of single-axis analyses of digital exclusion, we turn to the concept of intersectionality from feminist studies. Originally proposed by Crenshaw [26] to expose the marginalization of Black women under both sexism and racism, intersectionality stands against the tendency in critical social theorizing to treat individuals in independent categories. It emphasizes that systems of oppression are inherently bound together, creating singular social experiences for people who bear the force of multiple systems [27]. While IS research has explored the relationship between IT and identity [28,29], most studies focus on the individual or group level, investigating how IT mediates or shapes identities. However, an intersectional perspective views subjectivity as emerging from differential experiences produced by multiple and intersecting power structures [26]. This approach moves beyond a behavioral, individualistic sense of identity to one of the “social positioning” of individuals within social structures [30]. Applying intersectionality to digital health inequalities allows us to examine how various dimensions of disadvantage—such as age, language proficiency, and socioeconomic status—interact and compound to shape individuals’ experiences with digital health services.

#### Digital Capital Theory

The concept of digital capital by Ragnedda and Ruiu [31], an extension of the cultural capital theory by Bourdieu [32], offers another valuable lens for examining digital health disparities. Bourdieu [32] applied the idea of capital to signify the internal (eg, abilities and attitudes) and external (possessions and attributes) resources that people mobilize to achieve their goals in social life. He highlighted cultural capital as a form of capital that can be accumulated and transformed into other forms of capitals. Digital capital is made up of both digital competencies and digital technologies, which Ragnedda and Ruiu [31] argue is a form of capital in its own right and is essential for building up social, economic, and cultural resources in the digital world we live in today. Disparities involving digital skills originate in inequalities of access but are mediated by orientations that can only be understood in relation to total life contexts (eg, education, income bracket, age, location, and social support all influence a person’s access to digital technologies and the level of digital skills they can acquire) [33]. Digital capital is a relatively new concept that scholars have begun to explore empirically through various methodological approaches [34]. Digital capital may be estimated, for example, at the individual level by assessing a person’s digital literacy and skills, at the organizational level by measures of digital infrastructure (including the digital competence of personnel), and at the locality level in terms of the quality of the area’s IT infrastructure.

Digital capital theory points us to the hypothesis that traditional forms of capital (such as economic, cultural, and social) are converted into digital capital and vice versa and provides the conceptual tools to examine how and to what extent this occurs, thereby illuminating how social inequality relates to digital inequality. If digital spaces—due to social inequality and underlying power structures—become increasingly stratified, there will be significant impacts on how individuals from differing backgrounds gain accumulated forms of capital through the digital realm. In other words, digital capital theory offers an explanation as to why people who already face systemic inequities find that these disparities widen when services are digitalized.

Recent studies have begun to explore how digital capital interacts with other forms of capital to influence health outcomes and access to digital health services [35]. However, there remains a need for more in-depth, theoretically informed research on how digital capital intersects with other dimensions of disadvantage in shaping experiences with digital health technologies.

In the context of this study, intersectionality works as an overall guiding principle for understanding how people’s lives and characteristics stem from and lead to multiple axes of disadvantage, while digital capital theory helps us understand how these axes of disadvantage play out in terms of access to and use of digital resources.

### Research Gap and Objectives

Our previous narrative review [12] highlighted that while existing literature recognizes the multifaceted nature of digital inequality, there is a critical lack of in-depth, theoretically informed studies examining how different dimensions of disadvantage combine to affect digital health disparities. The review found the available literature on digital health disparities, particularly in relation to video consultations, to be sparse and primarily descriptive rather than explanatory. Most research has focused on identifying barriers and enablers without adequately exploring the complex interplay of factors contributing to these disparities.

Importantly, our review revealed no theoretically informed studies that examined how different dimensions of disadvantage combined to affect digital health disparities. This gap in the literature limits our understanding of how multiple disadvantages intersect and compound to shape individuals’ experiences with digital health services.

Building on these findings and responding to the recommendations of our narrative review, this study aims to address these critical gaps. By doing so, we seek to move beyond the descriptive accounts that have dominated the field and provide a richer, more nuanced theorization of digital health disparities. This approach allows us to explore the complex ways in which different aspects of disadvantage interact, compound, and manifest in the context of digital health services. Our goal is to contribute to the development of more inclusive and equitable digital health services by offering insights into the lived experiences of multiply disadvantaged individuals, as called for in our previous work.

This study represents a direct response to the research agenda proposed in our narrative review, aiming to deepen our understanding of digital health disparities and inform more effective, equitable strategies for digital health implementation.

## Methods

### Overview

This study used a qualitative, interpretive approach to explore the intersecting effects of age, socioeconomic status, and limited English proficiency on experiences with digital health services. The focus on age, language proficiency, and socioeconomic status was informed by our aforementioned narrative review [12], indicating these as key predictors of lower digital service uptake and barriers to access. We adopted narrative inquiry [36] as our primary methodology, which aligns with our aim to center the voices and experiences of marginalized patients. The study was conducted in Redbridge, one of London’s most diverse boroughs. It has >65% of its residents from Black and minority ethnic communities, predominantly Asian (42%). Over 90 different languages are spoken in the borough, with nearly a quarter of residents having a first language other than English. The borough includes several areas ranked among the 20% most deprived in England, with an unemployment rate (8%) exceeding London’s average. This demographic and socioeconomic profile made Redbridge an ideal setting to examine intersecting dimensions of disadvantage in digital health care access. Participants were recruited through the Redbridge Respiratory Service within the North East London NHS Foundation Trust, community organizations, and snowball sampling.

Inclusion criteria for participants were as follows: (1) aged ≥65 years, (2) limited English proficiency (self-reported or identified by health care providers), (3) living within an Index of Multiple Deprivation decile of 1 to 5, (4) having attempted ≥1 video consultation, and (5) residing within the Redbridge borough.

As this was a doctoral research project, the primary investigator, LH, conducted all interviews and primary analysis, with regular supervision and analytical discussions with her supervisory team to challenge interpretations and biases throughout the research process. Semistructured narrative interviews were conducted with 17 participants between July 2022 and January 2023. All (17/17 100%) study participants had access to health care through the NHS despite being multiply disadvantaged. The interview guide was designed to elicit rich narratives about participants’ experiences with digital health services, particularly video consultations. Questions explored their overall health care journey; experiences with digital technologies; and perceptions of how their age, language abilities, and financial situation affected their access to and use of digital health services. As a multilingual researcher fluent in Hindi, Urdu, and English, LH conducted all interviews directly in participants’ preferred languages. Participants could freely switch between languages during interviews, which many did. This linguistic flexibility allowed participants to express themselves more fully and comfortably when discussing their experiences. All non-English segments were translated to English during transcription by LH, while “broken English” was transcribed verbatim when the meaning was clear. In addition to interviews, LH conducted ethnographic observations with 9 (53%) of the 17 participants, including home visits and participation in daily routines. These observations provided valuable contextual insights into participants’ living conditions, family environments, and technological exposure. The ethnographic data were analyzed alongside interview transcripts to provide richer context to participants’ narratives and to understand how their home environment and daily routines influenced their experiences with digital health care.

LH used thematic narrative analysis [37] using an intersectionality lens to identify key themes while preserving the integrity of individual stories. The analysis process involved the following: (1) familiarization with the data through repeated reading of transcripts, (2) open coding to identify initial themes and patterns, (3) development of a coding framework informed by intersectionality theory, (4) axial coding to explore relationships between themes, and (5) selective coding to refine and integrate themes into a coherent narrative.

To enhance the intersectional analysis, a modified version of the Equity Design Collaborative’s meta-empathy mapping approach was incorporated [38]. This approach focuses on understanding users’ needs through transformative empathy, moving beyond surface-level observations to deeply understand how systemic barriers and power structures affect user experiences. This modified approach shaped the analysis in 3 key ways.

First, it guided us to examine both immediate barriers (ie, language difficulties) and deeper structural challenges (ie, how health care systems may inadvertently privilege certain cultural norms). Second, it helped identify power dynamics in health care relationships. Third, it informed how outputs such as user personas (discussed in subsequent sections) were structured to capture both individual circumstances and systemic influences on participants’ experiences. This methodological lens helped ensure findings reflected not only individual experiences but also broader systemic factors affecting remote care access.

Several strategies were used to ensure the trustworthiness of findings [39], including prolonged engagement with participants through multiple interactions, triangulation of data sources, member checking with participants to verify our interpretations, and maintaining a reflexive journal to document the decision-making process and potential biases.

Particular attention was paid to ensuring that participants fully understood the nature of the research and their rights, given potential language barriers and vulnerabilities. As researchers, we also acknowledge our own positionalities and how they may influence the research process. LH is a female Muslim of South Asian descent, which facilitated trust building with many participants but also required ongoing reflexivity to avoid assumptions based on shared cultural backgrounds. To synthesize findings, LH developed 4 [40] theoretically informed narrative portraits and user personas that captured key intersecting dimensions of disadvantage, including how age, language barriers, socioeconomic status, cultural factors, and health conditions combined to shape participants’ experiences with digital health care. These are reported in detail in our previous paper [40] and serve as complementary outputs that distill key themes into accessible archetypal stories, balancing the need to honor individual perspectives with extracting cross-cutting insights about the interplay of technology and disadvantage.

### Ethical Considerations

The study received ethics approval from the NHS Research Ethics Committee and the University of Oxford’s Central University Research Ethics Committee. Informed consent was obtained from all participants, and pseudonyms were used to protect their identities. Ethical approval was obtained from East Midlands—Leicester South Research Ethics Committee and UK Health Research Authority (September 2021, reference number: 21/EM/0170; Integrated Research Application System project ID 300719).

## Results

### Overview

The analysis revealed 8 key themes that illuminate how multiple dimensions of disadvantage intersect to shape experiences with digital health services, particularly video consultations. These themes highlight the complex ways in which the digitalization of health care can exacerbate existing inequities, erode trust, compound oppression, and reduce patient agency for multiply disadvantaged patient populations.

### Digital Interactions May Have a Weak Presence

The concept of “absent presence,” originally developed by Gergen [41] to describe technology-induced distraction in face-to-face interactions, takes on new significance in the context of digital health care. The analysis found that participants consistently reported a sense of diminished presence in digital health care interactions, particularly in video consultations. This weakness of presence manifested in 3 key ways: delayed responses, mechanical intonation, and perceived motionlessness.

Delayed responses were frequently noted by participants as a sign of disengagement. Maneshi, an Indian immigrant aged 83 years, articulated this experience as follows:

If my doctor is doing twenty other things on his side of the screen while I’m talking, even if he is technically listening, it just doesn’t feel like a genuine conversation to me...When I go to the GP [general practitioner] in person, those distractions aren’t there. I know the GP is looking at me and having a conversation with me.

This account illustrates how the perceived divided attention of health care providers during video consultations can erode the sense of a genuine, engaging interaction. The lack of immediate responsiveness disrupts the natural rhythm of conversation, leading to a feeling of disconnection.

Mechanical intonation was another aspect that contributed to the sense of weak presence. Fowzia, a recently widowed immigrant from Pakistan, expressed this concern as follows:

The absence of eye contact and non-verbal cues makes you feel...I don’t know it’s off putting. It feels like I’m talking to a computer program instead of a real doctor. They used to, the doctors, they would bring a lot of comfort and assurance; now, it’s replaced with a sense of disinterest. It just makes you think you know? Do they even care?

Participants described feeling as though they were interacting with a “computer program” rather than a real person, noting the flat, unemotional tone often used by health care providers during video consultations. Recent research in general practice has shown that video-mediated communication affects sensory conditions in clinical interactions, technical issues such as delayed facial expressions and sound can disrupt natural conversation flow, and the screen interface limits health care providers’ ability to read and respond to patient cues [42]. This lack of vocal vitality, as conceptualized by Stern [43], can significantly impact the patient’s perception of the provider’s engagement and empathy.

The third manifestation of weak presence was perceived motionlessness. Abed, a recently retired repairman aged 69 years, highlighted this issue as follows:

His movements don’t signal any interest. I mean I guess that could just be because I’m seeing his face and can’t tell over video call how much he’s actually moving. But I’m not seeing hand movements or his head moving much you know.

In face-to-face interactions, body language and subtle movements play a crucial role in conveying attention and engagement. However, in video consultations, the limited visual field and potential technical constraints can result in an appearance of stillness that patients interpret as a lack of involvement or interest. Basmah, a Bangladeshi immigrant aged 73 years with arthritis and limited mobility, noted the following:

Back in the day, a doctor’s touch and a comforting pat on the back meant so much. Now, it’s different, you just hear typing with those hands.

This triad of delayed responses, mechanical intonation, and perceived motionlessness collectively contributed to a sense of “absent presence” in digital health care interactions. Therefore, many (11/17, 65%) participants reported feeling disconnected and unheard during video consultations, potentially impacting the quality of care and patient satisfaction. Mohammad, a Sri Lankan immigrant aged 70 years with multiple chronic conditions and living with his extended family, starkly put the following:

Facial expression and body language is so important. When you talk to people you can see what’s wrong and things like that and some of the things you don’t know whether when the GP say things, how they mean it because I can’t tell over remote. I don’t think the whole system is fracturing, I think it’s completely collapsing.

While all patients may experience reduced presence in video consultations, this sense of disconnection is particularly problematic for patients with limited English proficiency, who rely heavily on nonverbal cues and physical presence to support communication and understanding. The technical barriers to presence can additionally compound existing language barriers, potentially leading to misunderstandings or missed clinical information.

### Digital Encounters May Weaken Relationships

Analysis revealed that digital encounters, particularly when there was no previous in-person relationship, often weakened the therapeutic relationship between patients and health care providers. Participants consistently expressed difficulty in forming bonds through screens, highlighting the importance of relational foundations for ethical care.

Rajpreet, a first-generation Indian woman experiencing economic hardship and multiple chronic conditions, articulated this challenge as follows:

Doing this by video makes it harder. If I met her in person, maybe we could connect more...but I know women like her, that’s not to say in a bad way, just that she doesn’t really struggle with the same things that I do, it’s very different when two women come from...I don’t know how to say...just that...she’s of a different social class...if that makes sense.

This quote illustrates how the lack of physical presence can exacerbate perceived social and cultural differences, making it more difficult to establish a connection and mutual understanding.

The importance of preexisting relationships was emphasized by many (14/17, 82%) participants. Fatima, an Afghan woman aged 81 years with diabetes and heart conditions, who relies on her children for translation, explained the following:

I think it [video consultation] was easier because I had met Dr. Samari before. So I already had that initial relationship with her. I’ve seen her a few times. I felt comfortable with her. I think it would have been a bit more awkward if it had been like a first meeting.

Conversely, participants who had video consultations with unfamiliar clinicians often reported fewer positive experiences. Ramnik, an Indian immigrant aged 78 years with hypertension and diabetes, who lives alone and relies on community support services, explained the following:

You stay in the online waiting room being all confused and then they let you into the call. And its someone you’ve never seen before. And he just wants yes or no. Then it’s finished. You can go. That’s it. You know? So I don’t know. I don’t like it. I don’t. This is why I don’t want to see the GP unless it’s really bad. When I did it [video consultation] with my own specialist it wasn’t like this. That time was good because he knew me and I knew him.

This account highlights how the lack of a prior relationship can lead to a sense of disconnection and dissatisfaction with the consultation process.

Some (5/17, 29%) participants stressed the need for occasional in-person visits to establish and maintain a connection:

It’d be nice if they could see a person like myself every three months or normal patients at least once every six months. Yeah, so you know, so then you know them. So that builds up some, like, friendship as well. Now there’s no friendship. But that’s why, you know, it’s important we get to know who the person is. Here there’s no chance to do that.

This suggestion underscores the perceived value of face-to-face interactions in building and sustaining therapeutic relationships.

The findings indicate that even when participants had successive video consultations with the same health care provider, they rarely developed a sense of building a strong and positive therapeutic relationship. This contrasts with face-to-face environments, where the patient-provider relationship typically strengthens with each encounter. Some (4/17, 23%) participants even described a deterioration in their relationship over repeated remote encounters, unless preceded by face-to-face meetings.

The weakening of therapeutic relationships through video consultations has consequences for marginalized older adults who often rely on trusted health care provider relationships to maintain engagement with health services. Limited English speakers face additional barriers to relationship building in digital settings, where language difficulties are compounded by technical constraints.

### The Shift From Relational to Distributed Continuity

The analysis also revealed a significant shift from relational to distributed continuity as digitalization increased after the COVID-19 pandemic. This transition often left patients feeling lost, unsupported, and struggling to navigate their care effectively. The loss of relational continuity was particularly pronounced for marginalized older adults who had previously relied on long-standing relationships with health care providers.

Tasneem, a Bengali immigrant aged 75 years, expressed this loss as follows:

Dr. Talib has seen me through so much. I could speak to her about anything, and she really listened and understood me.... [Now] I don’t even know my doctor’s name. How can I trust someone I don’t even know?

This quote encapsulates the profound impact of losing a trusted health care relationship and the challenge of building trust in a system of distributed care.

The fragmentation of care was a recurring theme among participants. Arjun, a retiree aged 72 years, described his frustration with the depersonalization of care as follows:

It’s becoming less and less personal. It’s like you are not a person there you are just a face. For example, the company I work for we went through the same sort of process, I joined in ’87 with BT and then in 1993 they brought this employee individual identification numbers they’re called right, so you’re given a nine digit number and then after that whenever you wanted to talk to the HR department or pay group or something like that, that’s the thing that you gave them and that’s it. So you only become a number, in this case you’re only becoming a face to the GP, always a new number, new face, new GP, it’s not who you are or what you are.

This shift to distributed continuity often resulted in communication breakdowns and potential risks to patient care. Priya, a Punjabi woman aged 71 years with diabetes, expressed her concerns as follows:

Ordering repeat medicine with the GP always main problem. They are, you know, taking so long and even sometimes they don’t know your condition there because communication, everything is so broken. They just forget everything. Like you are the new person to them. You know that’s the problem with the GP always.

They don’t know my history or me. I feel scared they’ll make a mistake with the dosage but what can I do?

The loss of community connections was another significant aspect of this shift. Another participant highlighted how digital triaging erased the familiarity and efficiency of previous care arrangements:

[B]efore, you know, they knew everything already. They knew you. And they had my history. Whereas. Where I am now, as I said, the first five minutes of any session are taken up with me explaining who I am, what my situation is. And then, you know, explaining what the problem is, why I’m seeing them all the rest. And this is repeating after filling out a whole econsult first. It’s a right shame.

The fragmentation of care extended beyond primary care to specialist services. Najma, a Pakistani immigrant aged 66 years with limited English proficiency and respiratory conditions, living in council housing, described her frustration with revolving specialists and poor communication:

Now my specialist has also now changed. Three of them I went through. At the moment one, I think one is they know she’s the main one. She was good, but now I don’t know which one. Last two weeks ago they giving me one medicine which is a high dose they asked me to stop and they my daughter she sent a lot of e-mail to her but she don’t reply anything here so this one [the new specialist] I think she’s not so good. Communication is not good. You keep chasing them, you know?

These experiences highlighted how the shift to distributed continuity has disrupted the holistic, coordinated care that many patients, especially those from marginalized communities, relied upon. The fragmentation of care across digital platforms has created new challenges in maintaining consistent, personalized health care relationships and effective communication between health care providers and patients.

### Digital Interactions May Compound Oppression

Findings revealed that digital health services often compounded existing forms of oppression and discrimination, particularly for participants with limited English proficiency and low digital literacy. These individuals faced multiple, intersecting barriers in accessing and navigating digital health platforms, which exacerbated their existing challenges in health care settings.

Rajpreet, a first-generation Indian woman aged 74 years living in council housing with multiple chronic conditions, articulated this challenge:

I told her, don’t mind me saying this Dr. Kaur, but you don’t really know what it’s like, you come from a different background or world whatever you want to call it [laughs], you know medically sure, but over video you can’t grasp it, you won’t understand because you don’t have it either, my condition, what surrounds it, that sort of thing...

This quote illustrates how digital interactions can amplify cultural and experiential gaps between patients and health care providers, making it more challenging to discuss sensitive health issues.

Language barriers were particularly problematic in digital settings. Samiyah, a Pakistani grandmother aged 69 years, with limited English proficiency and chronic pain conditions who recently moved in with her son’s family, noted the following:

It’s already hard to explain my symptoms in English, but over video, it’s even worse. I can’t use gestures or show them exactly where it hurts. Sometimes I feel like they don’t understand me at all.

Low digital literacy compounded these challenges. Tasneem shared the following:

I struggle with technology, and now I have to figure out how to use these apps just to see my doctor. It makes me feel stupid and left behind. Sometimes I just give up.

The shift to video consultations also highlighted existing inequalities in access to technology. Mohan, living on a basic pension in social housing with diabetes and heart disease, explained the following:

They tell us to do video calls, but I don’t have a smartphone or good internet. It’s like they’re saying healthcare is only for people who can afford fancy gadgets.

Participants from marginalized groups often found themselves at the intersection of multiple disadvantages—language barriers, cultural differences, low digital literacy, and limited access to technology—all of which were amplified with video consultations.

### Digitalization May Erode Trust in Health Providers and Systems

Findings revealed that the shift to video consultations also frequently eroded participants’ trust in health care providers and systems. This erosion of trust was often rooted in a sense of depersonalization and lack of continuity in care, particularly for marginalized and susceptible populations. Research has also documented how marginalized communities may have lower trust in health care providers due to several factors, including historical medical mistreatment, documented disparities in quality of care, and systemic barriers to culturally and linguistically appropriate services [44,45]. In this study, this broader context of health care mistrust appeared to be exacerbated by the shift to video consultations, particularly when patients could not establish consistent relationships with health care providers.

Hasan, with his multiple chronic conditions and previous negative health care experiences, articulated this sentiment strongly in the following manner:

You don’t wanna be a part of this system. Like you can’t trust these people in the NHS... Because I need the GP, but to be honest, I don’t trust that like you know, she’s not very good and she she doesn’t really care, right? That’s the impression I get so I try not to go but then it gets worse.

This quote illustrates how the perceived lack of care and attention in digital interactions can lead to a cycle of disengagement and worsening health outcomes.

The impersonal nature of video consultations was a recurring theme. As Fowzia explained the following:

The personal connection is not the same. I miss the stuff that didn’t require words, stuff you could just see and feel and the comfort of being physically there in the same room. It’s harder to build that trust through a screen.

This highlighted the importance of nonverbal cues and physical presence in building trust, which many felt was lacking in digital interactions.

The erosion of long-standing relationships with health care providers was particularly distressing for some participants. Fatima shared the following:

Thing is you can’t even think about trusting the GP now even if you wanted to this way [over video consults]. These things take a lot of time, beta, my old GP, I knew him for 10 years, every small flu, back ache, little cold he knew it all. I don’t even know if I have another 5 years left in me and if I keep seeing a different GP over a different platform where does that leave me, beta?

This account underscores how the fragmentation of care across multiple health care providers and platforms can disrupt the accumulation of shared knowledge and understanding that forms the basis of trust in health care relationships.

The erosion of trust sometimes led to nonadherence to medical advice. Zainab, a Pakistani immigrant aged 73 years with heart disease, who had previously experienced dismissive treatment from health care providers, explained the following:

I mean why should I take it [the medication], ok yeah they prescribe it but they don’t even bother listening to me, click clacketing away at their keyboard, not even looking at the screen, they think they know what’s wrong with me just like that? I don’t trust it one bit course I’m not gonna take it.

For some, the distrust in the health care system led to anxiety and avoidance. Hamida, a Bangladeshi mother aged 75 years living with her daughter’s family and experiencing both physical health conditions and growing anxiety about health care interactions, shared the following:

My daughter thinks I have anxiety because I don’t want to deal with the NHS. I don’t know, maybe I do. But to me it’s more about the fact that they can’t be trusted.

However, it is important to note that not all participants experienced an erosion of trust. In a notable disconfirming case, 1 participant reported high levels of trust in video consultations due to a preexisting, long-term relationship with her general practitioner. This suggests that strong, preexisting therapeutic relationships may buffer against the potential erosion of trust in digital interactions.

These findings highlight the complex relationship between digitalization and trust in health care. Video consultations can risk eroding the personal connections and continuity of care that many patients, particularly those from marginalized groups, rely on to build trust with their health care providers. This erosion of trust can have serious implications for patient engagement, adherence to treatment, and overall health outcomes.

### Digitalization Increases Complexity, Which May Lead to Disorientation

The analysis highlighted that the introduction of multiple digital platforms and access points often led to increased complexity and disorientation for participants. Many (12/17, 70%) struggled to navigate the various digital pathways and processes, leading to frustration and, in some cases, disengagement from health care services.

Mukesh, a man aged 85 years with cognitive challenges, articulated this complexity in the following manner:

The health system as a whole there is a lack of communication and sharing between the different functions. I mean I’ve got access to a long COVID clinic. Then I also deal with my GP and there should be some information exchange between the two. Not me filling them both in on my own. And then with the hospital, they’re not connected in a way that they can get anything from my GP to the hospital and it has been very frustrating.

This quote highlights how the fragmentation of digital systems can place an additional burden on patients, particularly those with complex or multiple health conditions.

The sense of being overwhelmed by digital options was a common theme. Ramnik expressed the following:

I felt like I was drowning in all the options, do I call the practice, do I do this econsult thing, do I use the NHS app, do I first check the website, but then the website it too complicated anyways and then I’m back to square one. It’s overwhelming, especially when you’re already struggling with other things, this is the last thing I should have to worry about.

This account illustrates how the proliferation of digital access points, while intended to improve accessibility, can paradoxically create barriers for some patients.

The disorientation experienced in digital health care settings was often compounded by language barriers and limited digital literacy. As Samiyah shared the following:

I wouldn’t know the first thing about doing a video call. He [son] set it up for me, clicked some stuff, and had it up and running and I just sat there. They did all the talking without me, but it probably was for the best anyways because I don’t know if I could’ve even said what I needed to properly, the language issue, the screen issue, just looking at it all was too much for me.

This quote underscores how digital health care can inadvertently exclude patients who lack the necessary language skills or technological proficiency, potentially exacerbating existing health inequalities.

The complexity of digital systems also led to challenges in maintaining continuity of care. Mohammad noted the following:

Look I’ve got more health issues than I can count on both of my hands, navigating through different services and providers, half remote, half in person, half on the phone, I just feel like I’m lost in a maze. I’m constantly juggling between appointments with different specialists, trying to piece together the whole story for myself and for the providers too because they themselves don’t know the full story. It’s exhausting and overwhelming.

This account highlights how the fragmentation of care across various digital and in-person platforms can create a significant cognitive and emotional burden for patients, particularly those managing multiple health conditions.

The disorientation caused by digital complexity was often exacerbated by socioeconomic factors. As Maneshi explained the following:

Not having the money to go private adds another layer of hard to the mix. I can’t afford the luxury of choosing the most convenient healthcare option. No. Instead, I’m forced to navigate through a patchwork of NHS resources that takes weeks, months, even years and it’s just getting worse because they’re trying to move things online now and my brain is already just scattered from long-covid first and then the mess that is the NHS trying to be something they aren’t so this is just the cherry on top.

This quote illustrates how the digitalization of health care, when not adequately supported or implemented, can compound existing health inequalities and create additional barriers for those already struggling to access care.

### Engagement With Digital Services May be Shaped by Previous Experiences of Racism and Discrimination

Findings revealed that participants’ willingness to engage with digital health services was often profoundly influenced by their previous experiences of racism and discrimination within the health care system. These past negative experiences created a foundation of mistrust that often extended to new digital health initiatives.

Hamida, with her growing anxiety, articulated this heightened vigilance as follows:

So I’m literally now I’m very vigilant. I will check every single medication. I will read the leaflet 3 times, I will Google it because I don’t trust these people they already prescribed the wrong one [medication] to me once before.

This quote illustrates how past negative experiences can lead to a deep-seated mistrust that influences future interactions with health care services, including digital platforms.

The intersection of racial identity and socioeconomic status in shaping health care experiences was highlighted by Abed in the following manner:

Well, yes. At the end of the day, yes, I do think that if I was a white person from a rich background, instead of brown and poor, I might have been treated differently.

This perception of differential treatment based on race and class extended to video consultations, with participants expressing concern that these biases would persist in digital interactions.

Some (9/17, 53%) participants described a resigned acceptance of interpersonal racism from health care staff:

You know, sometimes you can tell they’re a little bit... racist, but it’s OK, you know, it’s not a big deal.

And sometimes you get certain doctors who are racist. But it’s whatever, I’m used to that.

These statements reveal a troubling normalization of discriminatory treatment, which may influence patients’ expectations and engagement with digital health services.

Language barriers were identified as a particular challenge in video consultations, often intersecting with perceptions of racial discrimination:

They make it such that you know she’s not given one [appointment for his wife]. If you know what I mean. It’s I think it’s easier for them to treat people who, for example, who they can see is very much quite different. For example, if you have really great English and you’re able to communicate, you’re able to get your points across, you won’t deal with it [racism] as much as you will for example, if, it’s just ok.

This quote highlights how language proficiency can intersect with racial bias to create additional barriers to accessing health care, including digital services.

Some (6/17, 35%) participants, like Mukesh, expressed concern that video consultations might amplify existing biases:

Not too long ago my wife she had a video appointment because she had an issue with the hand. So the doctor like any questions we asked, he was just reluctant to answer and he had the, you know, the sarcasm.... So you just know where some doctors, you don’t know whether it’s racist or not, but like because we call it that because we feel that way.

This account suggests that the physical distance in video consultations may exacerbate perceptions of dismissive or discriminatory treatment.

The cumulative effect of these experiences led participants like Tasneem to express extreme distrust in the health care system:

This is what I mean. And that’s why I can’t trust these people with your life. You can’t. You can’t. You you’d rather die than trust some idiot with your life because you’re gonna die anyway.

This level of distrust poses significant challenges to the adoption and effective use of digital health services among marginalized communities.

### Digital Interactions May Reduce Patient Agency

The study revealed that the cumulative effect of the previously discussed factors often resulted in reduced patient agency. Many (14/17, 82%) participants, particularly those facing multiple disadvantages, felt disempowered and unable to effectively advocate for themselves in digital health care interactions:

I’m not very happy with the new GP, but I’m scared to change. The next one might be even worse.

This quote illustrates how the lack of options and fear of further negative experiences can trap patients in unsatisfactory care arrangements, reducing their ability to seek better alternatives.

The complexity of digital systems often led to confusion and reliance on others, as Najma described the following:

I can’t tell you how many times I’ve tried using the website or app and just ended up more confused. Which link was I supposed to click? How do I even describe my symptoms properly in writing? I don’t know half these medical terms they’re asking. My English is no good. Eventually I just give up and tell my son to figure it out for me instead.

This account highlights how language barriers and limited digital literacy can significantly undermine patients’ ability to independently navigate their care.

The lack of physical presence in video consultations was also cited as a factor reducing patient agency. Mukesh, who experiences complex neurological symptoms, shared the following:

I mean doctors have it tough now seeing us all through video calls. But it almost feels pointless for me. He’s just staring at notes on some other screen barely listening. I can’t really show him what’s going on with my body in a genuine way. And to be quite honest it just feels like he’s already decided before properly hearing me out.

This quote underscores how the limitations of video consultations can leave patients feeling unheard and unable to effectively communicate their concerns.

The reliance on family members further eroded the personal agency of participants like Samiyah, who explained the following:

At my age, it’s impossible to keep track of the different numbers to call or steps to do appointments online. My children handle everything now—they email test results, book consultations, order medications for me. I feel so helpless relying entirely on them, but I don’t really have much of a choice.

This dependence on others for health care management significantly diminishes patients’ autonomy and control over their own care.

Some participants, like Mohammad, felt entirely excluded from digital health care due to lack of access to necessary technology:

We don’t have good internet like that or one of those fancy smartphones they keep saying to use, you see that phone? [points to older Samsung phone with cracked screen] That’s what I have and it only works when we can pay for the you know [data]. My nephew always talking about this gadget and that, we just can’t afford it. I know they started some phone video service during corona, but it wasn’t for me. It feels like they are saying either we have to use this stuff or else we don’t deserve to get treatment.

This quote highlights how socioeconomic factors can create barriers to accessing digital health services, further reducing patient agency.

These 8 themes collectively illustrate the complex and intersecting ways in which the digitalization of health care can exacerbate existing inequities for multiply disadvantaged patients.

## Discussion

### Principal Findings

#### Overview

This study provides critical insights into how multiple dimensions of disadvantage intersect to shape experiences with digital health services, particularly video consultations, among older, low-income, individuals with limited English proficiency. The findings revealed a complex interplay of factors that contribute to digital health disparities, extending beyond notions of access and skills to encompass issues of presence, trust, continuity, oppression, complexity, and agency. The analysis revealed that the digitalization of health care can exacerbate existing inequities, erode trust, compound oppression, and reduce patient agency for multiply disadvantaged patient populations. Video consultations often created dynamics of “absent presence,” where patients perceived health care providers as distracted or disengaged, leading to a sense of disconnection. The lack of previous in-person rapport negatively shaped patients’ perceptions of subsequent video consultations with unfamiliar clinicians, which led to weakened relationships. The shift eroded established continuities of care for some, with the fragmentation of relational continuity becoming apparent. Digital interactions compounded experiences of oppression for patients navigating multiple, intersecting forms of structural disadvantage, such as age, ethnicity, and socioeconomic status. The complexity of navigating multiple digital platforms and pathways led to profound disorientation and fragmentation of care, especially for those with limited digital literacy or language proficiency. Experiences of racism and discrimination within health care settings shaped patients’ engagement with digital services, often leading to disengagement and mistrust. The cumulative effect of these factors resulted in a significant reduction in patient agency, particularly for marginalized individuals, undermining their ability to effectively navigate their care and make informed decisions.

The experiences of our participants suggest that digital health technologies, rather than being neutral tools, act as both mirrors and magnifiers of existing social inequalities. This aligns with the concept of the “digital poorhouse” by Eubanks [46], where technology reinforces and exacerbates existing patterns of marginalization. In this study, the shift to video consultations not only reflected existing disparities in health care access but also often amplified them, creating new barriers for those already struggling to navigate the health care system. This finding challenges the often-optimistic rhetoric surrounding digital health innovations [47,48]. While proponents argue that digital technologies can democratize access to health care [49,50], our results suggest a more nuanced reality. For multiply disadvantaged patients, the digitalization of health care services can create a cascade of exclusionary experiences, from difficulties in accessing technology to challenges in effectively communicating health needs in a digital environment.

#### Extending Intersectionality Theory in Digital Health Contexts

This research extends intersectionality literature by applying its insights to the study of digital health disparities, revealing specific mechanisms through which the increasing digitization of health care creates new forms of inequity and exclusion. The findings highlight how digital access disparities effectively excluded individuals with low digital literacy from video consultation, while limited English proficiency significantly reduced older adults’ engagement with health services in the digital space. Importantly, we found that digital competency and digital access do not always go hand in hand, demonstrating how digitalization, while improving health care access for some, simultaneously creates new vectors of exclusion that intersect with and exacerbate existing social inequities. Moreover, while earlier intersectionality research has focused primarily on traditional axes of oppression such as race, class, and gender, this study highlights the emergence of new vectors of disadvantage related to digital access, literacy, capital, and competency. This underscores the need for an expanded understanding of intersectionality that accounts for the growing centrality of digital technologies in shaping health outcomes and experiences.

This study shows that digital health disparities do not operate along independent axes of division but often overlap, interlink, and interact, demonstrating how patterns of dominance and vulnerability intersect to shape people’s experiences with digital health care. We argue that inequity and digital health exclusion are relational and occur along multiple fracture lines, which differentiate people’s spaces of opportunities, well-being, and level of agency. These disparities are produced and reinforced through complex social relationships and interactions within health care systems and broader societal structures. The COVID-19 pandemic may have brought out new instantiations and shed light on what was less visible before; however, the roots of digital health inequity are deeply entrenched in systems of power and social order.

#### The Constellation of Challenges for Multiply Disadvantaged Patients

The findings from this study revealed that video consultations created a constellation of challenges for older patients with multiple disadvantages. Participants described feeling profoundly disoriented in digital spaces, struggling to navigate unfamiliar platforms and processes. This disorientation was compounded by disrupted continuity of care, as they cycled between health care providers in fragmented digital encounters. The remote modality also engendered a weak sense of presence and connection—many (13/17, 76%) felt their health care providers were not fully attentive or did not understand their needs. Crucially, the digital interfaces exacerbated feelings of disempowerment and loss of agency. Participants felt adrift, unable to steer the direction of their care. Intersecting barriers reinforced the following: limited economic, social, and linguistic resources; social and cultural isolation; low digital, health, and health care system literacy; and physical impairments of illness and age. Some (9/17, 53%) perceived the digital challenges as yet another form of discrimination. These intersecting factors fed into a cycle of growing disengagement and mistrust toward individual providers and the health care system as a whole.

#### Digital Capital and Health Equity

Our results also contribute to the emerging literature on digital capital [35] by illustrating how disparities in digital competencies and access intersect with other forms of disadvantage to shape health outcomes. The struggles of our participants to navigate complex digital health systems reflect not only a lack of technical skills but also a broader deficiency in the social and cultural capital needed to effectively engage with digitalized health care. This finding aligns with the theory of capital conversion by Bourdieu [32], suggesting that disadvantages in 1 domain (eg, socioeconomic status) can translate into disadvantages in another (eg, digital health access).

#### Trust, Presence, and the Digitalization of Care Relationships

Our findings on the erosion of trust and the sense of “absent presence” in video consultations raise important questions about the nature of care relationships in digital environments. Drawing on the work by Giddens [30] on trust in modern societies, we can interpret these experiences as reflective of the disembedding of social relations that occurs with increased digitalization. The loss of physical copresence in health care interactions appears to disrupt established mechanisms for building and maintaining trust, particularly for patients who may already have reasons to distrust health care institutions.

This erosion of trust and presence challenges dominant narratives about the efficiency and convenience of digital health services. While video consultations may offer logistical benefits, our findings suggest they may come at the cost of the relational aspects of care that are particularly important for susceptible patients. This aligns with the critique of the logic of choice in health care by Mol [51], suggesting that the move toward digital health services may prioritize a transactional model of care over a relational one.

#### Compounding Oppression and Reduced Agency

Perhaps the most concerning finding is that digital health interactions can compound existing forms of oppression and reduce patient agency. This aligns with critical perspectives on technology that view it not as a neutral tool but as a social force that can reinforce existing power structures [52]. In the context of health care, where power imbalances between health care providers and patients are already pronounced [53], the addition of digital interfaces appears to further tilt the scales against marginalized patients. The reduction in patient agency observed in this study has important implications for patient-centered care and shared decision-making, which are increasingly recognized as crucial elements of high-quality health care [54]. Our findings suggest that current approaches to digital health may be undermining these important principles for certain patient populations.

#### Methodological Contributions

This research also makes significant methodological contributions to intersectionality literature by demonstrating the value of qualitative and narrative-based approaches to studying the lived experiences of marginalized groups. While quantitative approaches have dominated much of the existing research on digital health disparities, our research draws on feminist and critical race theories that emphasize the importance of storytelling and counternarratives as forms of epistemic resistance [55,56]. By using in-depth narrative interviews and persona development as key methodological tools [40], we provide a more nuanced and contextualized understanding of how digital health disparities are experienced and navigated by multiply disadvantaged individuals.

#### Implications for Practice and Policy

Viewed through the lens of health equity and social justice, our findings suggest that the rapid digitalization of health care risks exacerbating existing health disparities. This aligns with the concept of “digital redlining” proposed by Gilliard and Culik [57], where digital systems create new forms of discriminatory exclusion. The compounding of oppression and reduction in patient agency experienced by our participants raise serious concerns about the potential for digital health technologies to undermine principles of equity and patient-centered care.

However, these findings also point to potential avenues for intervention. Through reconditioning the intersectional nature of digital health disparities, policy makers and health care providers can develop more flexible, targeted approaches to support susceptible patients. This might involve not only addressing technical barriers to access but also working to build the broader forms of capital needed to effectively navigate digital health systems. For example, offering a range of communication options, including in-person visits, and providing additional support for patients navigating digital systems.

In addition, our results underscore the importance of maintaining and strengthening relational continuity in health care, even as care becomes increasingly digitalized. This might involve strategies to ensure patients can maintain relationships with preferred health care providers across digital and in-person interactions. Third, our findings point to the need for greater attention to issues of structural competency in the design and implementation of digital health systems. This could involve training health care providers in culturally competent digital communication and the development of digital health interfaces that are more inclusive and culturally sensitive. Finally, our results suggest that efforts to address digital health disparities must go beyond simply providing access and skills training to address deeper structural inequalities. This aligns with calls for a “digital determinants of health” framework that recognizes the broader social, economic, and political factors shaping digital health equity [58].

### Limitations and Future Research

While this study offers valuable insights, several methodological limitations warrant consideration. The relatively small sample size (N=17) and geographical confinement to Redbridge, London, limit the generalizability of our findings. Our reliance on narrative interviews may have introduced recall and social desirability biases, potentially skewing participants’ accounts of their digital health experiences. The inclusion criterion of having attempted ≥1 video consultation may have inadvertently excluded those facing the most severe barriers to digital health access. In addition, our study focused primarily on video consultations, potentially overlooking other forms of digital health interventions.

Looking ahead, several key areas warrant further investigation to address these limitations and expand our understanding of digital health equity. Longitudinal studies are needed to track the long-term impacts of digital health services on marginalized populations, providing insights beyond the snapshot our study offers. Intervention studies should evaluate targeted approaches to address identified barriers, such as building digital capital and fostering trust, which could help overcome some of the access issues noted in our limitations.

To address the geographical limitations of our study, comparative analysis across different health care systems and cultural contexts could identify transferable principles and context-specific challenges in promoting digital health equity. This broader perspective would enhance the generalizability of findings and inform more universally applicable strategies.

From a policy perspective, there is a pressing need to examine how existing health policies and digital strategies impact health disparities. The study analysis could provide valuable context for understanding the systemic factors influencing digital health equity, beyond the individual experiences captured in our study.

Finally, to complement this study’s qualitative insights and address the limitations of our small sample size, future work should focus on developing and validating quantitative measures for intersectional digital health disparities. This would enable population-level tracking and more comprehensive evaluations of digital health interventions, providing a broader evidence base to complement in-depth qualitative studies like our study.

These research directions will contribute to a more nuanced understanding of digital health equity and inform evidence-based strategies for inclusive health care digitalization. By addressing the limitations of this study and expanding the scope of the investigation, future research can build a more comprehensive picture of the challenges and opportunities in promoting equitable access to digital health services.

### Conclusions

This study provides critical insights into how the digitalization of health care can deepen disparities for older, low-income, individuals with limited English proficiency. By applying an intersectional lens to the study of digital health inequalities, our research reveals the complex, overlapping, and mutually reinforcing nature of digital exclusion. Our findings underscore the need for intersectional approaches to digital health equity that address the multifaceted nature of disadvantage.

This study makes several unique contributions to the field. First, it extends the application of intersectionality theory to digital health disparities, demonstrating how multiple dimensions of disadvantage interact to shape experiences with digital health services, particularly video consultations. This approach has revealed nuanced insights into how different forms of marginalization compound to create unique barriers to accessing and benefiting from digital health innovations.

Second, our development of theoretically informed user personas [40], grounded in intersectionality and digital capital theories, offers a novel methodological approach for representing the complex lived experiences of multiply disadvantaged patients. These personas provide a powerful tool for humanizing research findings and informing patient-centered service design in digital health contexts.

Finally, by centering the voices of marginalized patients, our research exposes how the rapid shift to video consultations has inadvertently exacerbated existing inequities and eroded trust for some susceptible groups. This challenges prevailing narratives about the universally positive impact of digital health innovations and highlights the need for more nuanced, context-sensitive approaches to digital health implementation.

Furthermore, our findings on the erosion of trust, the sense of “absent presence” in digital consultations, the shift from relational to distributed continuity of care, the weakening of patient-provider relationships, the compounding of oppression, the increased complexity leading to disorientation, the influence of previous experiences of discrimination on engagement, and the reduction in patient agency contribute new insights to the ongoing discourse on the impact of health care digitalization.

In conclusion, as health care continues to digitalize, it is imperative that we remain vigilant to the unintended consequences of technological change and work to ensure that the benefits of innovation are equitably distributed. This will require a fundamental rethinking of how we design, deploy, and evaluate digital health interventions, as well as a renewed commitment to the principles of social justice and health equity.

## Abbreviations

IS: information system
NHS: National Health Service
